# Introducing machine learning model to response surface methodology for biosorption of methylene blue dye using *Triticum aestivum* biomass

**DOI:** 10.1038/s41598-023-35645-z

**Published:** 2023-05-26

**Authors:** Sheetal Kumari, Anoop Verma, Pinki Sharma, Smriti Agarwal, Vishnu D. Rajput, Tatiana Minkina, Priyadarshani Rajput, Surendra Pal Singh, Manoj Chandra Garg

**Affiliations:** 1 Amity Institute of Environmental Sciences, Amity University Uttar Pradesh, Sector-125, Noida, 201313 Uttar Pradesh India; 2 School of Energy and Environment, Thapar Institute of Engineering and Technology, Patiala, India; 3 Department of Hydrology, Indian Institute of Technology Roorkee, Roorkee, 247667 Uttarakhand India; 4 Department of Electronics and Communication Engineering, Motilal Nehru National Institute of Technology Allahabad, Prayagraj, 211004 Uttar Pradesh India; 5 Academy of Biology and Biotechnology, Southern Federal University, 344090 Rostov-on-Don, Russia; 6 Surveying Engineering Department, Wollega University, Nekemte City, Ethiopia

**Keywords:** Environmental sciences, Hydrology

## Abstract

A major environmental problem on a global scale is the contamination of water by dyes, particularly from industrial effluents. Consequently, wastewater treatment from various industrial wastes is crucial to restoring environmental quality. Dye is an important class of organic pollutants that are considered harmful to both people and aquatic habitats. The textile industry has become more interested in agricultural-based adsorbents, particularly in adsorption. The biosorption of Methylene blue (MB) dye from aqueous solutions by the wheat straw (*T. aestivum*) biomass was evaluated in this study. The biosorption process parameters were optimized using the response surface methodology (RSM) approach with a face-centred central composite design (FCCCD). Using a 10 mg/L concentration MB dye, 1.5 mg of biomass, an initial pH of 6, and a contact time of 60 min at 25 °C, the maximum MB dye removal percentages (96%) were obtained. Artificial neural network (ANN) modelling techniques are also employed to stimulate and validate the process, and their efficacy and ability to predict the reaction (removal efficiency) were assessed. The existence of functional groups, which are important binding sites involved in the process of MB biosorption, was demonstrated using Fourier Transform Infrared Spectroscopy (FTIR) spectra. Moreover, a scan electron microscope (SEM) revealed that fresh, shiny particles had been absorbed on the surface of the *T. aestivum* following the biosorption procedure. The bio-removal of MB from wastewater effluents has been demonstrated to be possible using *T. aestivum* biomass as a biosorbent. It is also a promising biosorbent that is economical, environmentally friendly, biodegradable, and cost-effective.

## Introduction

Textile dyeing plant industries produce a significant amount of waste, 5% of which ends up in wastewater effluents of about 637.3 million cubic metres per year, which contributes significantly to the pollution of water bodies^1^. Wastewater from industries that make dyes and pigments, as well as many others, is typically rich in colour and organic material. The use of dyes is widespread in sectors like textiles, rubber, paper, plastic, and cosmetics. Textiles are the first among these several industries in the use of dyes to colour fibre. Dye discharge from textile industries causes severe air, water, and soil pollution and thus adversely impacts the environment. The textile industry has recently grown to be a significant problem that has an impact on both people and the environment^2^. Wastewater that contains dye is hazardous because it contains toxic substances, suspended solids, and other chemicals^3,4^. A chemical that results from their interaction is extremely dangerous for people, plants, and aquatic life. The result is waterborne diseases^5^. MB is the most common and popular dye in the textile industry, used to colour wool, silks, and cotton. MB is a positively charged anionic quinonoid structure and the chemical formula of MB is C_16_H_18_ClN_3_S. Methemoglobinemia, tissue necrosis, mental confusion, and vomiting are all possible side effects of MB toxicity^6^. Limiting oxygen transfer and preventing sunlight from reaching water bodies are two negative effects of dyes on the environment^7^.

Recently, several reports on dye removal methods have been released^8^. The three main treatment categories for the methods that were presented are chemical, biological, and physical treatments^9,10^. Some of the remarkable methods that are typically reported include adsorption, biological treatment, electrochemical treatment, advanced oxidation (AOP), and membrane filtration^11,12^. Pre-proofing is used to get rid of the dye. Each technique has advantages and disadvantages. The approach that is most frequently used is adsorption^13^. It enables the removal of pollutants at levels ranging from low to high. As a result, numerous studies have been carried out to create adsorbent materials that are efficient and affordable^14^. The most adaptable and widely used of these techniques is biosorption it is both affordable and user-friendly^15,16^. Numerous studies have supported and confirmed the use of a variety of materials for pollutant biosorption to remove contaminants^17,18^. Popular and highly effective biosorbents such as activated carbon are also more expensive^19^, which has led leading many researchers to search hunt for biosorbents that were inexpensive and easily accessible locally^20,21^. To remove the dye MB from textile wastewater, *T. aestivum* is used as a low-cost biosorbent in this study. It is a frequently discarded agricultural waste product that is readily accessible and can no longer be used for beneficial purposes^22,23^. Additionally, it is freely available or extremely inexpensive, making it a readily available and cost-effective biosorbent. The disadvantages of the synthesized adsorbents for the treatments of the dye wastewater are regeneration of the biosorbent is expensive and results in the loss of materials, require high dosage, and is economically non-viable for some industries like paper and pulp.

Numerous variables, including pH, the amount of biosorbent used, and dye concentration, can affect dye removal by biosorption^24^. The response surface method is a flexible computational method that can be used to optimise the biosorption process. It is beneficial for planning the modelling and enhancing the optimization of the experiments. It was formerly employed to simulate the biosorption procedure^25,26^. In this study, we use RSM’s central composite design (CCD) to optimise the biosorption of MB dye on *T. aestivum*. The proposed study aims to use *T. aestivum* as an economical and environmentally friendly biosorbent to remove the MB dye from the aqueous sample. The treatment of wastewater from the textile industry has been covered in a few research articles^7,27^, but most of them describe the current adsorbate in-depth and focus on metals or dyes. They talked about the many low-cost adsorbents available for treating wastewater from the textile industry.

**Figure 1:**
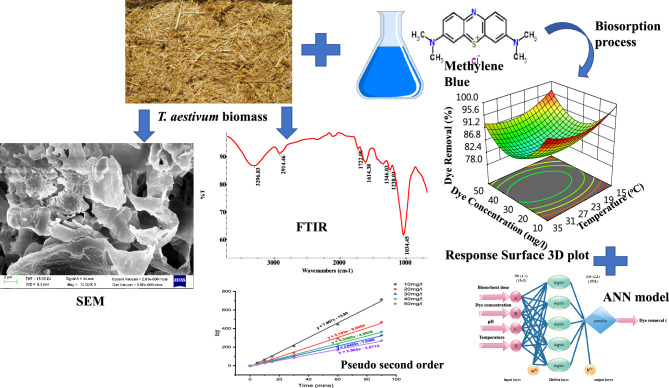
A schematic diagram of biosorption study.

Additionally, combining the RSM with machine learning (ANNs) could increase the reliability of the model. The emergence of better modelling methods with improved model performance, such as ANN, offers a substitute for polynomial regression. The output responses obtained by the CCD were compared to the predictions made by machine learning (ANN) using the MATLAB programme. A closer look at the literature, reveals several gaps and shortcomings. Previous research typically focussed on only investigated the thermodynamics, kinetics, and isotherms study of the biosorption process. These studies warrant a better understanding of biosorption, but there is still a great deal of work to be done along with designing of experiments (RSM) and machine modelling. This article provides a new novel approach to optimization modelling using RSM to investigate and examine how biosorbent dosage, initial dye pH, concentration, and temperature affect the critical behaviour of biosorption. The thermodynamic parameters, isotherms, biosorption kinetics and surface modification of *T. aestivum* were all determined by analysing the biosorption data using Fourier transform infrared spectroscopy. A schematic diagram of the biosorption study is shown in Fig. 1.

## Materials and methods

### *Preparation of T. aestivum biosorbent T. aestivum* is procured from neighbourhood vendors and employed in this investigation as a biosorbent

It is first divided into smaller pieces and repeatedly rinsed with water to remove residues any. The biosorbent is then placed and exposed to the sun to dry for one week. The dry biosorbent was then mortar-crushed. Using a sieve shaker, the ground biosorbent is sieved to keep uniform-sized particles, prior to being dried for 24 h in a hot air furnace at 70 °C, the particles that made it through a 250 µm sieve are used deionized water to rinse three times. Biosorption tests are conducted using the prepared biosorbent. The chemical composition of *T. aestivum* is listed in Table 1.

**Table 1:** The chemical composition of *T. aestivum.*

Constituent	Concentration (wt%)
Ash	7.9
Carbon	42.9
Chlorine	0.17
Hydrogen	5.7
Nitrogen	0.62
Sulphur	0.16
Calcium	0.18
Silica	4.5
Phosphorous	0.05
Lignin	11–22.9

The biosorption procedure is created to recognise biomass activation by employing FTIR spectroscopy to assess the functional groups on the biosorbent surface. With an experimental range of 400–4000 cm^−1^, the spectra are obtained using a PerkinElmer FTIR instrument.

### Preparation of stock solution of MB dye

In this investigation, experimental biosorption is conducted using MB (C_16_H_18_ClN_3_S xH_2_O) of analytical grade. The characteristic of MB is listed in Table 2.

**Table 2:** Characteristics of MB.

Particulars	MB
Molecular structure	
Chemical formula	C_16_H_18_ClN_3_S
Molecular weight	319.85 (g/mol)
Class	Cationic thiazine dye
λ max (nm)	664
Colour index name	Basic Blue 9 (BG9)
CAS number	61-73-4

The requisite quantity of MB dye powder and deionized water are combined to produce the stock solution of MB (500 mg/L). The given concentration levels are produced from the stock MB solution. It was utilised to gather the experimental results.

### Biosorption studies of MB on *T. aestivum*

The biosorption process parameters must be optimised according to variables including the biosorbent dosage, contact time, temperature, pH solution, and initial dye concentration to achieve maximal biosorption. Each experiment involved shaking Erlenmeyer flasks with 100 mL of MB dye solutions at 120 rpm for 60 min. When utilising a pH metre, 1 M NaOH or 1 M HCl solution was added to dye solutions to change the pH. The biosorbent was removed from the mixture using filter paper at intervals of 0.45 µm while stirring. An absorption spectrophotometer (Make: Labman Scientific Instruments Pvt. Ltd., Model: LMSP UV1900) is used to compare the dye concentrations before and after treatment. The means of each experiment's two runs were presented as concentrations. To examine the impact on biosorption kinetics, the test solutions' starting colour density and response time were changed. With the use of a pH metre (Make: HANNA instruments, USA, Model: HI 991001), the pH of the dye solution was changed. To determine how temperature affects different thermodynamic parameters, biosorption research was conducted using diluted HCl or NaOH solutions. Biosorption levels (q_t_) at time t (mg/g) were calculated using Eq. (1)^31^

1 $${q}_{t}= ({C}_{o} - {C}_{t}) V / (W )$$

C_t_ (mg/L) indicates the overall dye concentration, C_o_ (mg/L) the initial dye concentration, V the solution volume (L), and W the dry biosorbent mass (g). In order to determine the amount of biosorption at equilibrium, q_e_(mg/g), was shown in Eq. (2)

2 $${q}_{e} = ({C}_{o} - {C}_{e}) V / (W)$$

In this equation, C_e_ is the equilibrium dye concentration (in mg/L). To respond to the RSM investigation, the dye removal percentage was calculated using Eq. (3)

3 $$Dye\,removal\,(\%) = ({C}_{o} - {C}_{e})\,/\,({C}_{o}) \times 100$$

### Design of experiments using RSM

Because only one variable is changed while the other variables are kept constant in a standard experiment, the researcher ignores the synergistic effect of the components. A variety of optimization methodologies have been developed in operational analysis over the years, resulting in a long history of optimization studies^32^. RSM is a methodical statistical methodology that improves the agreement of the minimal test runs when evaluating the relationship between design responses and factors^33^. The quadrilateral design is provided because the CCD contains only a subset of the experiments required for the five-step factorial and provides schemes with the required statistical properties^34,35^.

Biosorbent dose (0.5–2.5 mg), initial metal ion concentration (10–50 mg/L), initial dye pH (4–8) and temperature (15–35 °C) make up the design criteria for the CCD of biosorption^24^. With two chosen as the axial levels, each variable had five levels: 1, 0, + 1, − α and + α. The study's selected independent variables are listed below, and their values and ranges are in Table 3.

**Table 3:** Codes and levels of independent variables used in the RSM model.

Code	Factors	Ranges and levels				
		− α	− 1	0	+ 1	+ α
A	Biosorbent dose (mg)	0.5	1	1.5	2	2.5
B	Dye concentration (mg/l)	10	20	30	40	50
C	Dye solution pH	4	5	6	7	8
D	Temperature (°C)	15	20	25	30	35

The number of experiments needed for CCD design can be determined by

4 $$N = 2n + 2n + nc$$

where c is the number of centre-point replicas, n is the number of numerical components, and N is the total number of experiments^25^. The graphical analysis, regression analysis, and experimental design were all carried out using software from Stat-Ease Inc. known as Design Expert. A total of 30 trials were designed, each containing six repetitions of the centre points, eight replications of the axial points, and sixteen replications of the cubical points, in accordance with Eq. (4). Regression equations were used to determine the variables' ideal circumstances. Using a four-point combination of four variables and three phases, the maximum organic sorbent dosage, pH, initial metal ion concentration, and temperature were all calculated^35^. This design was chosen because it met most of the criteria for optimizing bio-absorption studies^26^. Finding ideal process working conditions to meet performance standards is the primary goal of RSM.

### Modelling of the biosorption isotherm

We used the biosorption facts of MB on *T. aestivum* to solve the isotherm equations. According to the Langmuir isotherm, neither the target molecules nor the adsorbent surface will ever interact. The model also includes a restricted number of energetic websites, which are typically organised in a monolayer^36^. Langmuir isotherms may be used to simulate the biosorption process.

5 $$qe= \frac{{q}_{max} bCe}{1 + bCe}$$

q_e_ is the quantity of dye absorbed at equilibrium in mg and q_max_ is the maximum quantity of dye that can be absorbed via means of biomass in mg, C_e_ is the equilibrium MB awareness expressed in mg/L, whilst b is the Langmuir isotherm constant. Instead, the Langmuir equation's linearized form can be shown as follows.

6 $$\frac{1}{{q}_{e}} = \frac{1 }{{q}_{m}} + \frac{1}{{C}_{e}} {q}_{m}b$$

C_e_ denotes for equilibrium concentration of MB (mg/L), q_e_ for the quantity of MB absorbed at equilibrium (mg/g), and q_m_ for maximum/monolayer biosorption capacity (mg/g) respectively. The basic characteristics of the Langmuir isotherm are described by a non-dimensional dissociation constant, R_L_.

7 $${R}_{L} = \frac{1 }{(1+ b.{C}_{e})}$$

### Biosorption kinetics modelling

The experimental data on biosorption in this study were optimised using pseudo-first-order. This kinetics study used different dye concentrations (C_o_ = 10, 20, 30, 40, and 50 mg/L) to evaluate the kinetics for five to ninety minutes. The basic description of the biosorption rate determined by biosorption capacity is given below in accordance with Lagergren's first-order rate equation. Typically, a linear expression for this rate is used^37^.

8 $$ln \left({q}_{e}- { q}_{t}\right)= ln\,{q}_{e} - {K}_{1}t$$

The quantities of MB adsorbed on *T. aestivum* at equilibrium (q_e_) and at any time (q_t_), respectively; K_1_ (min^−1^) is the pseudo-first-order biosorption rate constant (q_t_). H^o^ suggested an expression-based rate-based kinetic model with quadratic coefficients has been put forth for the biosorption of dissociated metal ions (adsorbents) in coal particles. The adsorbent’s biosorption capacity is consistent with this model^38^. This model is consistent with the adsorbent's capacity for biosorption. The model presents a pseudo-quadratic rate equation and aims to separate the kinetics of the biomass concentration-based quadratic rate equation from the solvent concentration-based data. The pseudo-quadratic model's linear form is as follows in Eq. (9)

9 $$\frac{1 }{{q}_{t}} = \frac{1 }{{K}_{2}{q}_{e}^{2}}+ \frac{1 }{{q}_{e}}t$$

*T. aestivum* absorbs MB dye at equilibrium (mg/g) and at any time, which is designated as q_e_ and q_t_, respectively. The equilibrium rate constant for pseudo-second-order biosorption is K_2_. To determine how they would affect H^o^'s proposed pseudo-second-order model of biosorption kinetics, the test solutions' initial colour density and reaction time were altered.

### Biosorption thermodynamics studies

Thermodynamic parameters include entropy ($\Delta$S), changes in Gibb's free energy ($\Delta$G), and enthalpy ($\Delta$H), of biosorption at various temperatures for MB dye onto the *T. aestivum*^29^. Five different temperatures were used to investigate the impact of temperature on batch-by-batch tests of MB dye on *T. aestivum*. The following diagram iillustrate the thermodymanics parameters influences on $\Delta$G variation during the biosorption process^39^. The slope and intercept of the following function were used to calculate the change in entropy and enthalpy during the biosorption process.

10 $$\Delta G = \Delta H {-} T\Delta S$$

### ANN-based predictive modelling

**Figure 2:**
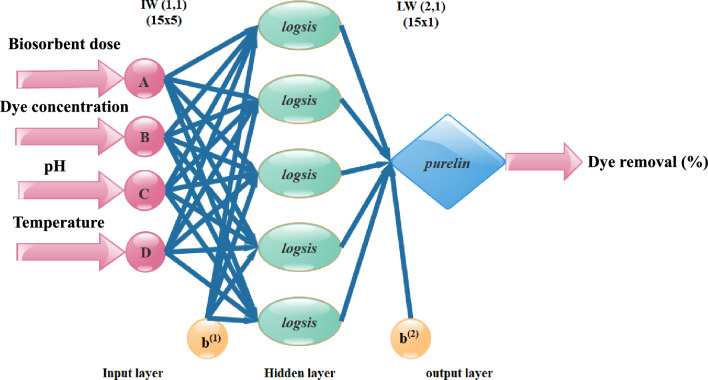
Structure of the ANN model for the dye removal % (MB) outputs.

Few studies have previously used machine learning (ANN) modelling to forecast dye removal of MB performance^40^. The neurons that make up an ANN are highly coupled processing units that have summing junction and transfer functions. ANN modelling, in contrast to RSM, includes an input (factors), target (experimental response), and output (predicted response). The input layer (representing independent variables), output layer (representing dependent variables), and hidden layers that link inputs with outputs are the layers in which the artificial neurons are placed^41^. Figure 2 illustrates the pattern of neuronal.

### Consent to participate

This research does not involve human participants and/or animals.

## Results and discussion

### Response surface methodology

The highest dye removal observed and anticipated MB biosorption values, and the matrix of experimental design are listed in Table 4. In 60 min, 30 tests in total were conducted. *T. aestivum* had the highest dye removal rate (96%) compared to other combinations with 1.5 mg of biosorbent, 10 mg/L dye solution, pH 6, and a temperature of 25 °C. The link between the independent variables chosen and the biosorption of the MB dye is described by regression equations, which are used to express RSM. For this investigation, the regression equation is expressed in terms of coded values is shown as Eq. (11).

11 $$\begin{gathered} {\text{Dye Removal}} \left( \% \right) = 84.778 + 3.026 * A + - 0.951042 * B + 0.74097 * C \hfill \\ + - 0.843281 * D + 0.150187 * AB + 0.0474576 * AC + - 0.732874 * AD \hfill \\ + - 0.0578228 * BC + - 0.164211 * BD + 0.340879 * CD + - 0.12876 * A^{2} \hfill \\ + 2.29724 * B^{2} + 0.286968 * C^{2} + 0.926208* D^{2} \hfill \\ \end{gathered}$$

**Table 4:** Actual value and projected MB removal are included in the experimental design matrix**.**

Run	A (mg)	B (mg/l)	C	D (°C)	Dye removal (%)	
					Actual value	Predicted value
1	10	40	7	20	84.93	84.60
2	20	20	5	30	89.00	89.39
3	15	30	6	15	90.00	90.17
4	10	40	5	20	84.00	84.01
5	20	20	7	30	91.77	91.76
6	10	20	5	20	85.43	85.77
7	15	30	4	25	84.28	84.44
8	5	30	6	25	78.00	78.21
9	15	30	6	25	84.66	84.78
10	20	40	5	20	92.06	91.74
11	15	10	6	25	96.00	95.87
12	15	30	6	25	84.94	84.78
13	20	40	7	30	90.00	89.72
14	20	20	5	20	93.26	92.89
15	10	20	7	30	87.00	87.38
16	10	40	7	30	84.36	84.73
17	25	30	6	25	90.59	90.31
18	10	20	7	20	87.00	86.59
19	20	40	5	30	87.16	87.57
20	10	20	5	30	85.59	85.20
21	10	40	5	30	83.12	82.78
22	15	30	6	35	87.03	86.80
23	15	30	6	25	84.81	84.78
24	20	20	7	20	93.51	93.90
25	15	30	6	25	84.45	84.78
26	15	30	6	25	84.72	84.78
27	15	30	8	25	87.64	87.41
28	20	40	7	20	92.11	92.51
29	15	50	6	25	92.00	92.06
30	15	30	6	25	85.09	84.78

Annotation: A (biosorbent dose), B (dye solution pH), C (initial dye concentration), and D (temperature).

**Figure 3:**
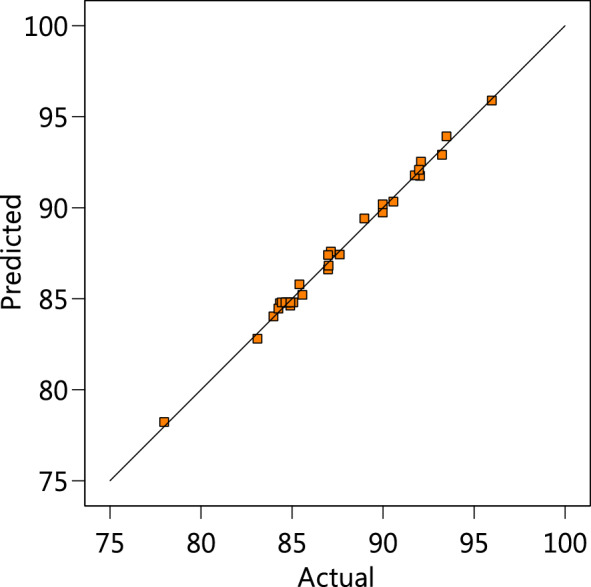
The expected values for MB biosorption are plotted against the experimental data.

A, B, C, and D are the coded variables used in this RSM investigation. To forecast how each element will react to different phases, the equation can be utilised in conjunction with the coded variable. The standard notation for superior and subordinate status is + 1 and 1, respectively. Using this coding equation, the relative effects of the variables are ascertained after comparing the coefficients of the factors. Figure 3 illustrates that the predicted value of MB adsorption is plotted against the actual value from data, yielding an R^2^ value of 0.9945, which validates the models’ accuracy and can be used in the experiment.

### ANOVA analysis

ANOVA analysis used all the experimental findings for the full factorial response variable that was duplicated at the central and axial points (Table 5). A significant quadratic model contribution is shown in the ANOVA findings in Table 5 with a p-value of less than 0.01. The significant model in the current investigation is shown by the sample F-value of 193.32. This huge F-value may be caused by noise with a mere 0.01% probability. The values obtained using R^2^ = 0.9945 show a strong correlation between the experimental data currently available and the predicted values of the model put forth to describe the property of the polynomial model. This correlation is described by the calculation of the coefficient, the mean deviation across the model described, and the values themselves. The results with R^2^ = 0.9945 show that there is a strong correlation between the experimental data that is currently available and the predicted values of the model that is suggested to reflect the property of the polynomial model. The determination of the coefficient, the mean deviation throughout the described model, and the value all demonstrate this link. The value of F is 4.54 shows that there may be a 5.43% risk that the considerable prevalence of Fit F-value deficiency is because of noise, and the absence of Fit is not statistically significant.

**Table 5:** ANOVA for biosorption of MB removal.

Source	Sum of squares	df	Mean square	F-value	p-value
Model	445.94	14	31.85	193.32	0.0001
A	219.76	1	219.76	1333.74	
B	21.71	1	21.71	131.74	
C	13.18	1	13.18	79.97	
D	17.07	1	17.07	103.58	
Lack of fit	2.23	10	0.2226	4.54	0.0543
Pure error	0.2451	5	0.049		

Std. Dev = − 0.4059, R^2^ = 0.9945, Adj. R^2^ = 0.9893, Predicted R^2^ = 0.9706.

**Figure 4:**
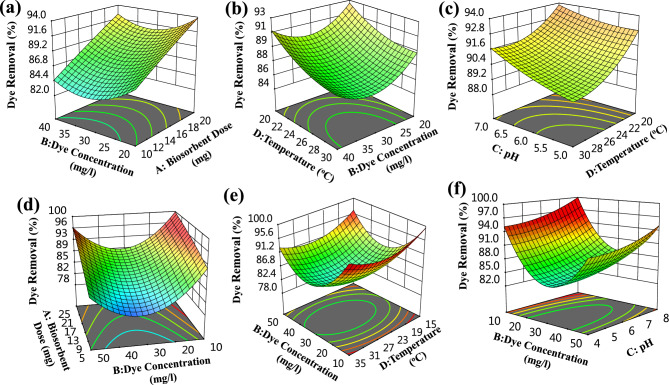
MB biosorption by *T. aestivum* biomass in a three-dimensional surface plot, illustrating the interactive effects of the four factors under test.

Response surface plots show the MB biosorption efficiency (%) response to common parameters based on most values of alternative parameters for a certain set of components is shown in Fig. 4a–f. These 3D plots' curves demonstrate how the process variables interact. The optimum scenario and interacting outcomes of the four evaluated factors are shown in the 3D aspect plots in Fig. 4a–f.

#### Effect of biosorbent dose

The availability and cost of biomass are the main deciding considerations when adopting it for large-scale industrial purposes. Biomass is one of the most exciting categories of biosorbents^39^. In terms of getting rid of heavy metals from wastewater, agricultural biomass has a whole lot of benefits, which include being a cost-powerful renewable natural biomass, having an excessive metal elimination efficiency, having an excessive ability for absorption, and being capable of regenerating and reusing the biomass^42^. Figure 4d demonstrates that the removal effectiveness of MB increases with increasing biosorbent dose and declines with decreasing dye concentrations. The primary cause for the simultaneous rise in MB's biosorption capability and an increase in biosorbent concentration is the availability of many more open active sites on the surface of the biosorbent^43^. On the other hand, Vijayaraghavan et al. discovered that the concentration of *T. aestivum* biomass increased together with the rate of MB biosorption from an aqueous solution^44^. The reality that the dye's biosorption per cent decreases as biomass attention increases demonstrates that the wide variety of dye molecules required to absolutely cowl all the lively adsorption sites in the biomass at excessive sorbent doses is insufficient^1^.

#### Effect of MB concentrations

The relationship between the biosorbent dose and the concentration of MB dye is shown in Fig. 4a. The MB removal percentages increase with the increase of biosorbent dose and dye concentration. The biosorption process is also influenced by the initial MB concentrations. Growing the preliminary dye concentrations usually causes growth within the elimination percentage. The biosorption quantity of dye on the surface of adsorbents increases as the initial concentration of MB increases^45^.

#### Effect of initial pH

The biosorption procedure may be motivated with the aid of using numerous variables, along with pH, preliminary concentration, and biosorbent dosage. Figure 4c describes the association between the pH and the temperature. While the initial pH level, MB concentration, and contact duration were retained at their zero levels, the three-dimensional surface plots (3D) in Fig. 4c show the simultaneous effects of pH and temperature on MB removal (%), respectively. The process of contaminant biosorption has been discovered to be most affected, among other things, by the initial pH level. pH levels influence a variety of processes, including the chemistry of metal solutions, the activity of functional groups in biomass, and the net charge on the surface of sorbent cells. Heavy metal ions and H^+^ may compete with one another for cellular active sites on the surface of biosorbent cells since the biosorption method for significant metals is usually potential of hydrogen ion concentration dependent^46^. According to the study of experimental findings, the *T. aestivum* biomass can more efficaciously soak up the MB dye because the pH rises, with maximum biosorption happening at approximately pH 8. The *T. aestivum* surface appearing as a biosorbent and the protonation and deprotonation of the MB dye can each be used to provide an explanation for the outcome.

#### Effect of temperature

The biosorption process sensitivity to temperature can be used to determine a biosorbent sorption capacity. The impact of temperature on the removal of Basic Blue 41(BB41) through effective microorganism-primarily based total leaf compost was assessed at various temperatures between 25 and 45 °C^47^. The outcomes of the experiment showed that a rise in temperature would result in a greater capacity for dye sorption (Fig. 4b). Figure 3e demonstrates that the slightly increasing the concentration at lower temperature the efficiency of dye removal also increases. Researchers have found that increasing temperatures increase the rate of solute diffusion, which has a significant impact on the sorbent's ability to absorb solutes^48^. However, the impact of temperature on biosorption is quite delicate and might be slightly increased at lower temperatures. The ability of the dye molecules to sustain contact with the biosorbent surface sites and the expansion of pore size with rising temperature were cited as the causes of this outcome. In general, a rise in temperature accelerates the rate of solute diffusion, which has a significant impact on the ability of biosorbents to bind to solutes^48^.

### Isotherms model for biosorption

**Figure 5:**
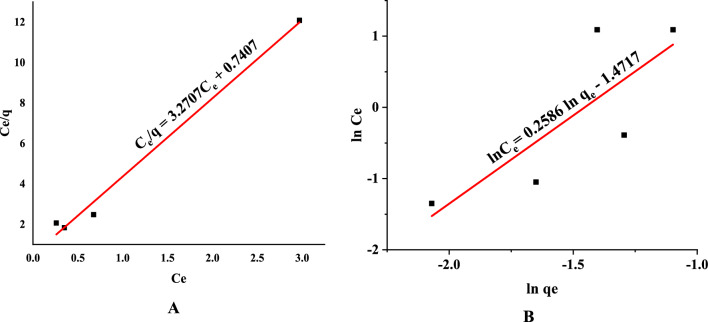
(**A**) Langmuir isotherm model and (**B**) Freundlich isotherm in linear form.

A fitting result of a linear line with a (C_e_/q) intercept to the Langmuir equation is displayed as (C_e_/q) versus (C_e_) shown in Fig. 5A. According to Table 6, The Langmuir isotherm's determined correlation coefficients were 0.9381. The biosorption's deviation from linearity is considered when calculating the second Langmuir constant, R_L_. In the current investigation, the equilibrium value of the dimensionless factor value, R_L_, which ranges from 0 to 1, was 0.062 (Table 6), indicating favourable biosorption. That confirmed that *T. aestivum* and MB had favourable biosorption (Fig. 5A).

**Table 6:** Parameters of Isotherm models of MB onto *T. aestivum.*

Isotherms	Statistical parameters	Values
Langmuir Isotherms	R^2^	0.9381
	q_m_ (mg/g)	0.3
	B (L/mg)	4.41
	R_L_	0.062
Freundlich Isotherm	R^2^	0.6391
	K_f_	0.22
	n	3.86

Figure 5B illustrates the values of 1/n and K_f_ determined from the intercept and slope of the linear plot of ln q_e_ versus ln C_e_^49^. The desired constants are provided with the regression equation as shown in Table 6. The favourable nature of biosorption was proved by the fact that n is between 0 and 1^50^. The Langmuir and Freundlich biosorption isotherms best explain the equilibrium results, demonstrating that monolayer formation mediates biosorption on a homogeneous surface**.** Figure 5B shows a linear fit of the Freundlich equation using a line with an intercept of ln K_f_ and a slope of n^49^.

#### Kinetic studies

**Figure 6:**
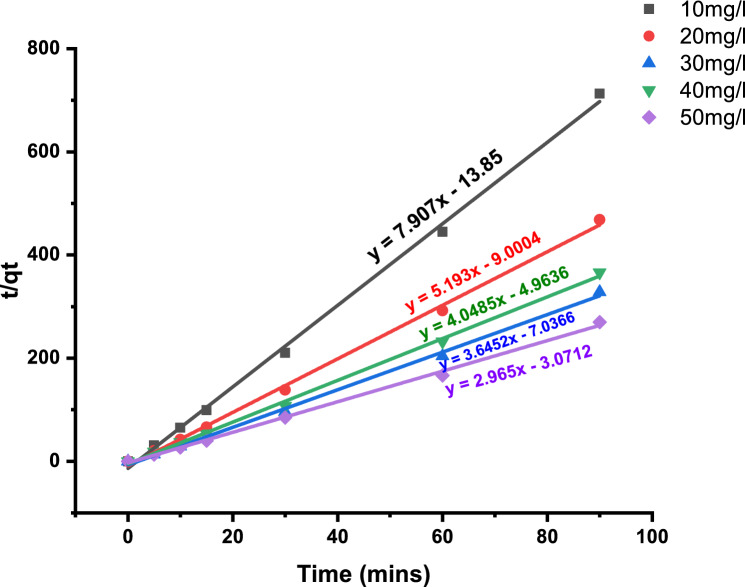
Pseudo second order kinetic curve for MB elimination% by *T. aestivum.*

The first-order kinetics' calculated K_1_, q_e_, and R^2^ values are shown in Table 7. As shown in Fig. 6, pseudo-second-order graphs were made by plotting t/q_t_ vs. time. The second-order rate constants have been calculated using the charts. The second order's calculated K_2_, q_e_, and R^2^ are supported by Table 7.

**Table 7:** Kinetic models of pseudo first and second order.

C_o_ (mg/l)	Pseudo I order			Pseudo II order		
	q_e_ (mg/g)	K_1_ (min^−1^)	R^2^	q_e_ (mg/g)	K_2_ (min^−1^)	R^2^
10	0.79	− 0.00267	0.9574	0.12	− 4.51409	0.9978
20	0.79	− 0.00266	0.9496	0.19	− 2.99619	0.9978
30	0.77	− 0.00309	0.9341	0.27	− 1.88837	0.9975
40	0.88	− 0.0012	0.6777	0.24	− 3.30213	0.9981
50	0.87	− 0.00139	0.8163	0.33	− 2.86242	0.998

Pseudo-second-order kinetics' correlation coefficients are becoming close to unity in contrast to pseudo-first-order kinetics. Therefore, it is evident that the pseudo-second-order model represents a biosorption that is more successful.

#### Thermodynamics study

As expected, the biosorption capacity of MB onto *T. aestivum* increases substantially while the temperature rises from 20 to 40 °C. The biosorption capability of *T. aestivum* is boosted through the biosorbent’s expanded pore length and the warming of the sorbent's surface. Raising the temperature causes the big dye molecule to penetrate more deeply, which also enhanced the large dye ion's potency, which lessens the impact of swelling^16,51^. As a result, MB was able to absorb the *T. aestivum* more quickly at high temperatures. Gibbs free energy ($\Delta$G), enthalpy ($\Delta$H), and entropy ($\Delta$S), among other thermodynamic characteristics, have all been calculated for the extrusion^28^. Furthermore, Table 8 also provides $\Delta$H, $\Delta$G, and $\Delta$S values for 20 mg/L preliminary MB dye concentrations.

**Table 8:** Parameters for the thermodynamic removal of MB from *T. aestivum.*

Temperature (K)	∆G (kJ/mol)	∆H (kJ/mol)	∆S (kJ/mol K)	Ea (J/mol)	S* (J K/mol)
288	− 9374.01	− 12,300.04	− 10.11	− 0.15	0.998
293	− 9385.16				
298	− 9240.83				
303	− 9253.67				
308	− 9228.04				

The negative values of ΔG demonstrated the spontaneity and viability of the adsorption process for MB sorption on *T. aestivum*. Because there is less unpredictability at the solid/liquid interface when MB is adsorbing to *T. aestivum*, the value of entropy ΔS (− 10.11 kJ/mol K) is negative. The negative value of ΔH (− 12,300.04 kJ/mol for MB) supports the exothermic character of the reaction. Good interaction between *T. aestivum* and MB is indicated by high levels of ΔH. This led us to the conclusion that the sorption of the dye in T. aestivum is a process of chemical biosorption.

### Sticking probability

The sticking probability (S*) is a function of the adsorbate/biosorbent system under discussion, but it is temperature dependent and needs to fulfil the criterion 1 < S* < 1 for optimum biosorption. The value of sticking probability was calculated from experimental data. It was calculated using a modified Arrhenius- type equation.

12 $$S^{*} = \left( {1 - \theta } \right)e_{ RT}^{ - Ea}$$

The parameter S* represents the measure of an adsorbate’s capability to persist on the adsorbent indefinitely. The surface coverage ($\theta$) at different temperatures was calculated to assess the effects of temperature on the sticking probability over the temperature range from 288 to 308 K. The slope and intercept of the ln (1 − ϴ) against 1/T plot can be used to determine the value of Ea and S*. The negative value of Ea shows that methylene blue dye removal by adsorption onto *Triticum aestivum* is favoured by a lower solution temperature, and the biosorption process is exothermic in nature. This biosorbent has affinity for methylene blue, indicating that it is a superior biosorbent for removal of methylene blue, as shown by MB sticking probability of S* < 1 on the surface of biomass is presented in Table 8.

#### Artificial neural networks (ANNs) modelling

ANNs are used to generate new processes, analyse existing ones, and anticipate the result and performance of systems^26^. The feed-forward neural network's optimal topology consists of an output layer, a hidden layer, and four neurons each in the input and hidden layers (including one neuron).

**Figure 7:**
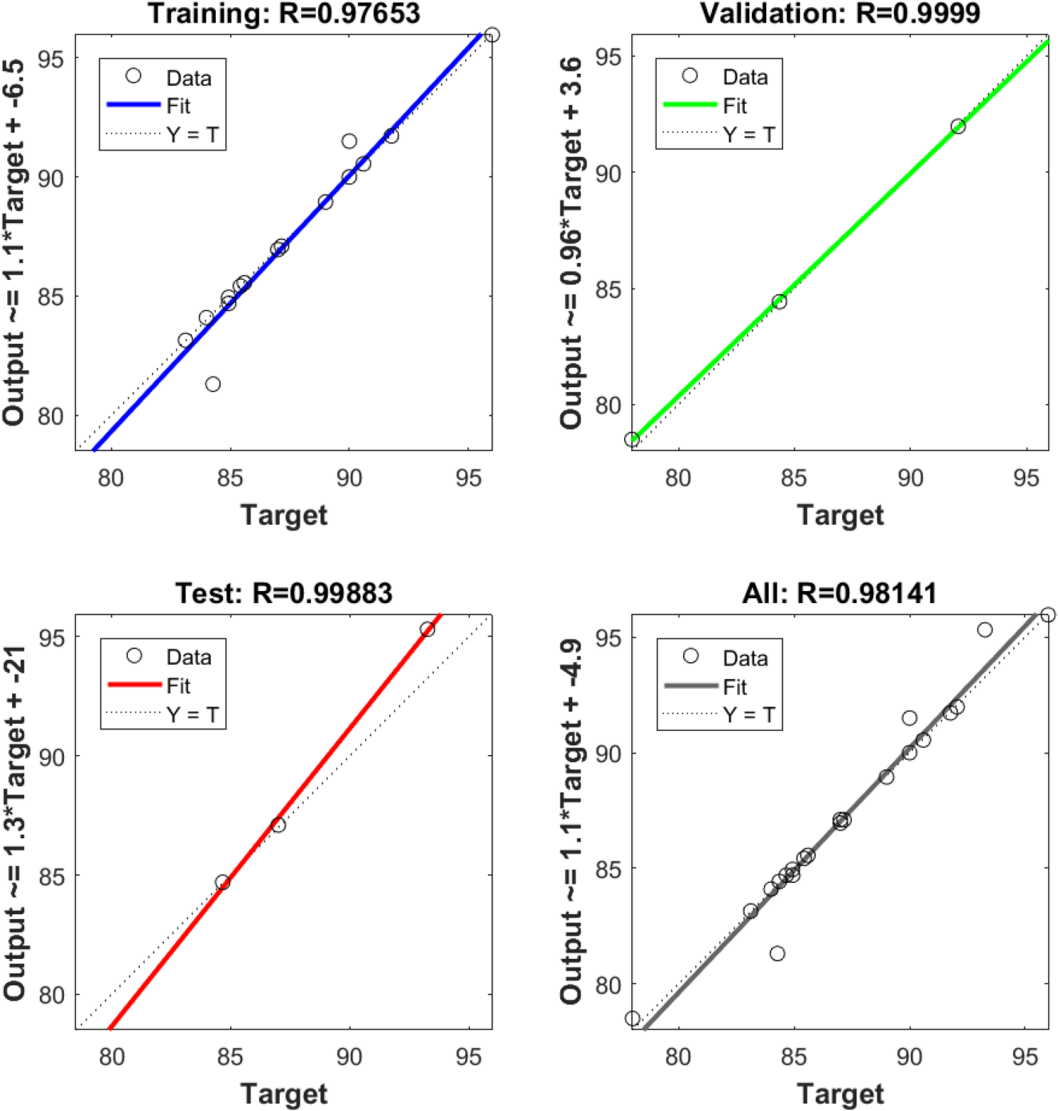
Regression plot for ANN of MB on *T. aestivum* training, validation, and prediction test.

**Figure 8:**
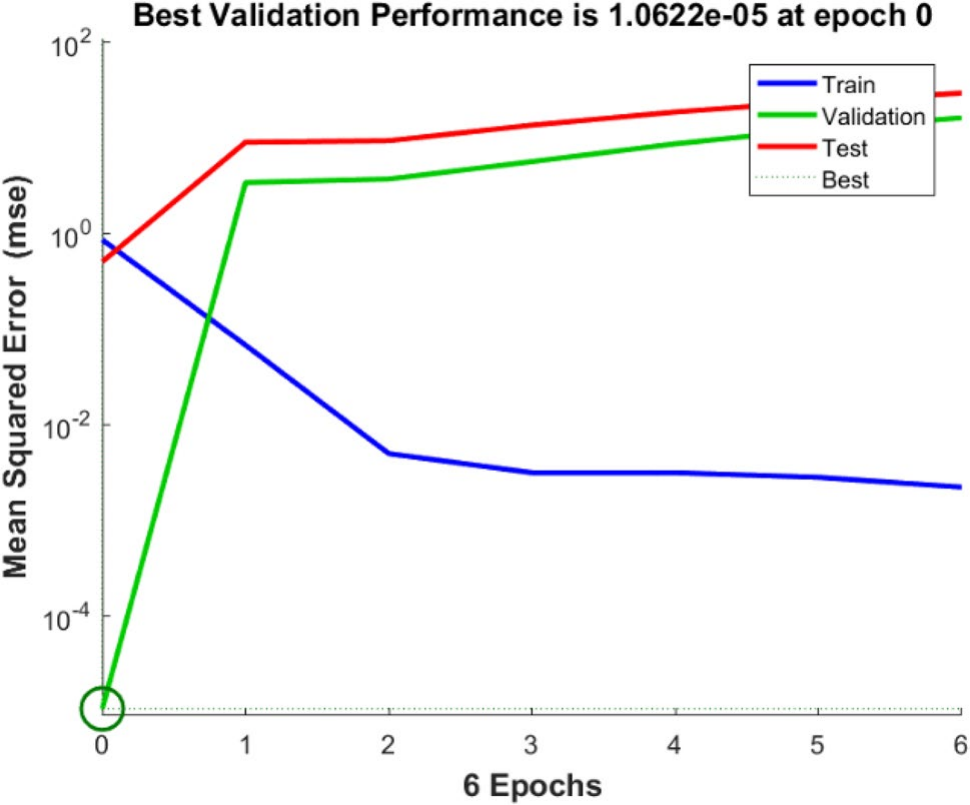
Performance of training, validation, and test error for ANN of MB on *T. aestivum.*

The experiments designed by the CCD provided the input and output for training. After training, a neural network’s weights and biases are displayed in Table 9. The model’s logsig (log-sigmoid) transfer function provides the necessary information for anticipating the outcomes. Figure 7 displays the expected values of the ANN model. In terms of the number of learning epochs, Fig. 8 analyses the ANN model's training, validation, and tests.

**Table 9:** Network weights and biases using the optimal parameters for the ANN model.

IW (1,1)	Input weight matrix (from input 1 to hidden layer 1)	[− 2.9914	2.0506	− 2.7009	3.1198;
		− 0.2268	− 3.4661	− 0.1809	− 3.9339;
		− 0.9184	− 1.7984	3.3151	− 4.2393;
		4.0913	− 1.3403	0.58331	− 3.553;
		− 1.243	− 3.8064	0.47701	3.6985;
		− 3.176	− 0.8716	− 1.7803	− 3.5459;
		4.4583	0.33737	0.46681	3.5853;
		2.9215	− 3.2848	0.80144	− 3.1329;
		− 1.147	− 3.5479	3.0714	3.033;
		− 4.6221	3.2439	− 0.6903	− 0.9442;
		− 1.2045	− 4.8009	− 2.1221	1.9949;
		− 0.6861	3.3286	3.6996	1.3839;
		0.67619	1.9046	− 4.2266	4.2753;
		− 3.8095	− 0.6657	3.8271	2.8507;
		1.1301	1.119	4.3342	− 1.8559]
b (1)	Bias to layer 1	[5.5598;	5.6217;	3.5416;	
		− 2.9443;	2.411;	2.826;	
		1.1548;	− 0.4262;	0.84572;	
		0.46688;	− 2.9484;	− 3.5026;	
		2.6258;	− 4.0129;	6.0707]	
LW (2,1)	Layer weight matrix (from hidden layer 1 to output layer 2)	[1.0019	− 1.9775	1.711	
		− 1.7747	− 0.545	0.9707	
		1.4694	1.0308	− 1.0613	
		− 1.5757	1.5921	− 0.1697	
		− 0.8304	− 0.1689	0.8036]	
b (2)	Bias to output layer 2	[− 0.440]

The RSM- predicted improved process conditions are also assessed using an ANN model. Biosorbent dose (2 mg), dye concentration (20 mg/l), dye solution pH (7) and temperature (20 °C) are used as input parameters for the ANN model. When the test error is lowest and the mean squared error has not changed for at least 1000 iterations, the training is terminated. The network is trained in this analysis for a total of 6 epochs. When the anticipated values from the ANN and RSM models are compared, it becomes evident that the values predicted by both models are considerably closer to the experimental results (Table 9).

#### Multiple response optimization

The experimental findings were optimised using Design-Expert software^35^. The 93.51% biosorption efficiency was attained under the ideal conditions shown in Table 10^25^ and conducted a special batch experiment to demonstrate optimization under ideal circumstances for comparison under suitable circumstances between projected and actual results. The difference between the projected value and the actual value is 93.90% or 93.51% confirming that the anticipated and actual values are the same, yielding verified model results. According to the requirements listed in Table 10, model equation is developed to maximise MB removal efficiency. Predicted numerical optimization was obtained 2 mg biosorbent dose, 20 mg/L concentration, 7 pH and at 20 °C temperature with 93.90% MB dye removal efficiency. The percentage error between RSM prediction and optimized condition is 0.41% and error between RSM and ANN is 2.17%. Validation experiment conducted as same input value gave 93.51% MB removal efficiency & projected reactions are consistent with model predictions validated under these ideal process condition (Table 10).

**Table 10:** RSM and ANN predictions optimization conditions for multiple responses.

Input variables	Optimized value	Removal efficiency		
		RSM prediction	Validation experiment at RSM optimized conditions	ANN Prediction at optimized conditions
Biosorbent dose	2.07 mg			
Dye concentration	20.02 mg/l	93.90%	93.51%	91.47%
pH	6.98			
Temperature	29.8 °C			
		**Percentage error**		
Between RSM prediction and validation		0.41%		
Between RSM Validation and ANN Prediction			2.17%	

#### Characterization of biosorbent

##### Surface modification analysis by FTIR

**Figure 9:**
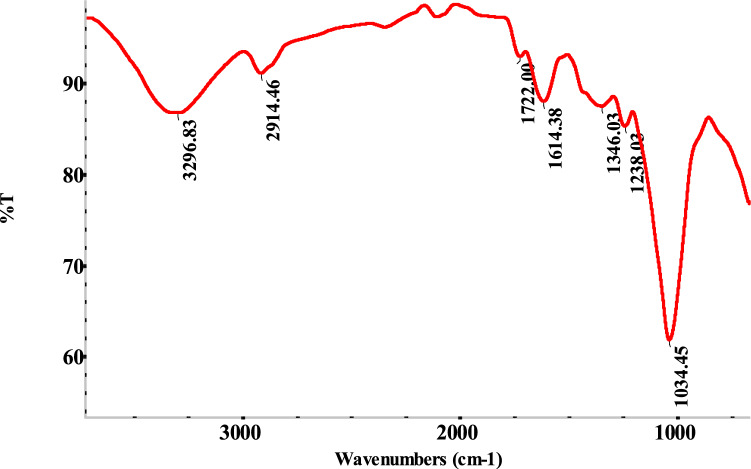
FTIR spectroscopy for *T. aestivum* biosorption of MB.

Using FTIR spectroscopy, surface alteration may be found, allowing the biosorption mechanism to be examined. Using the Perkin Elmer FTIR system, the FTIR spectrum data was Gathered. The surface of the biosorbent is visible with functional groups such as nitro, hydroxyl, carbonyl, carboxylic, phenol, and phenol groups in Fig. 9. FTIR spectra can be used to distinguish between the many functional groups that are present in biosorbent formations^52^. Two distinct peaks at 1372 and 1371 cm^−1^ and 1512 and 1511 cm^−1^, respectively, indicate the stretching vibration of the nitro-N–O groups, which were discovered to have been extended due to biosorption on biosorbent. The 1634 and 1632 cm^−1^ peaks are the stretches of C=C. The C–O stretch of various moieties and the carboxylic group have been implicated as the cause of the numerous strong, sharp peaks that were observed between the levels of 1100 and 1330 cm^−1^^24^. The hydroxyl functional group's O–H stretching vibration and the band at 3200–3600 cm^−1^ had previously been linked (Fig. 9). The stretching of the carboxyl groups in C=O is responsible for the peak around 1700–1800 cm^−1^. The surface charge differential may change due to positive or negative surface charges depending on the pH of the solution^2,6,53^. In a solution with a lower pH value, the system will operate more frequently and develop a positive surface charge. The hydroxyl group is indicated by the height increase at 3340 cm^−1^ caused by the MB absorbed on *T. aestivum*, as well as by the prolonged robust sharp top at 1034 cm^−1^. Peaks in the range of 1327–1372 cm^−1^ were caused by the interaction of MB and Nitro companies in *T. aestivum*^54–56^.

##### SEM evaluation

**Figure 10:**
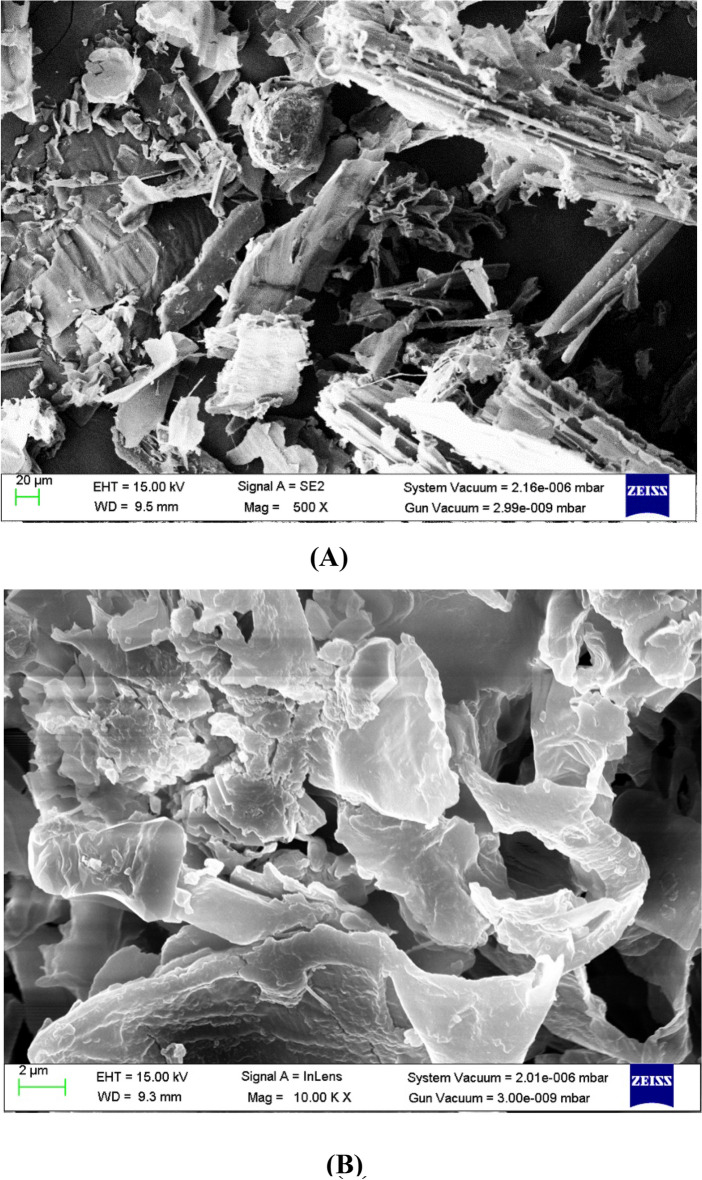
SEM images illustrated *T. aestivum* biomass: (**A**) before and (**B**) after MB biosorption.

The surface topography and properties of *T. aestivum* can be directly scanned using a scanning electron microscope (SEM) examination. SEM images are displayed Before and after MB biosorption, the biomass of *T. aestivum* (Fig. 10A,B). The biomass made of untreated *T. aestivum* had a rough and irregular surface, as seen in Fig. 10A. The look of fresh, shining particles absorbed on the surface of *T. aestivum* was depicted in Fig. 10B. Another distinguishing quality had been demonstrated (Fig. 10B). The surface area of polymeric *T. aestivum* has been reduced due to possible cross-linking between positively charged ions and negatively charged chemical functional groups in the cell wall. The surface of *T. aestivum* is rough and undulated, which increases the surface area exposure of the active biosorption sites and leads to MB's enhanced bio-absorption efficacy.

## Conclusion

The biosorption of MB dye onto *T. aestivum* was demonstrated in this work using the experimental variables of biosorbent quantity, dye pH, temperature, and concentration. The experimental result of the biosorption performance of MB was examined and found to be superior to the use of the CCD-primarily based RSM optimization technique and ANN. Using isotherms, kinetics, and thermodynamics studies, the best RSM results were examined. This study assessed *T. aestivum* ability to remove MB dye from wastewater. The experimental results were demonstrated to be closely related to the Langmuir isotherm model, which has a maximum biosorption capacity of 0.36 mg/g. Additionally, MB sorption on *T. aestivum* was studied using pseudo-second-order kinetics at a rate constant of (2.56 gmg^−1^ min^−1^). Thermodynamic analysis shows that the adsorption process is exothermic and spontaneous. After characterising the biosorbent by evaluation of the *T. aestivum* FTIR spectra, it was determined that the change of dye ions with counterions, which are typically attached to the surface through carboxyl, hydroxyl, and nitro groups, is the mechanism behind the metal binding. *T. aestivum* is a more affordable alternative adsorbent even if compared to commercial activated carbon, it has a higher capacity for biosorption. The use of *T. aestivum* as an adsorbent to take the colour out of water could be inexpensive and efficient.

## Data Availability

All data generated or analysed during this study are included in this published article.
